# Data on residential nearly Zero Energy Buildings (nZEB) design in Eastern Europe^☆^

**DOI:** 10.1016/j.dib.2022.108419

**Published:** 2022-06-28

**Authors:** Shady Attia

**Affiliations:** Department UEE, Sustainable Building Design Lab, Faculty of Applied Sciences, Université de Liège, Belgium

**Keywords:** New construction, Energy performance, Energy efficiency, Renewable energy, Thermal comfort, EPBD

## Abstract

This data article includes a dataset developed between 2020 and 2022 to characterize and analyze the state of energy efficiency of nearly zero energy buildings in ten Eastern European countries. The data article refers to the paper' Overview and future challenges of nearly zero-energy building (nZEB) design in Eastern Europe' (Attia and Kosinski et al., 2022). The data provides an overview of the status of building energy use, energy savings, and regulations for the newly constructed building stock, including Bulgaria, Croatia, Czech Republic, Estonia, Hungary, Latvia, Lithuania, Poland, Romania, and Slovakia. The importance of the dataset lies in its unique approach of data collection that provides detailed information on electricity, gas, oil, coal, and wood used in the investigated countries between 2015 and 2020. The methodology involves compiling and fusing data from national registries and depositories written in ten different national languages. Thus, the data is not available in Eurostat or any EU platform. The methodology followed to produce the data is mainly a literature review of restricted national publications and an extensive questionnaire with 14 national experts involved with the nZEB implementation plans and policies. The data include several parameters, including building energy efficiency thresholds expressed in the form of primary energy use intensity; primary energy conversion factors; renewable energy shares; building envelope performance requirements; mechanical ventilation performance requirements; thermal comfort requirements; construction rates of residential buildings; and heat pumps market penetration expressed as heat pump units per 100 households. The data paper is valuable for scientists to conduct future research to implement energy efficiency measures and renewables towards energy-neutral buildings.

## Graphical abstract

**Figure:**
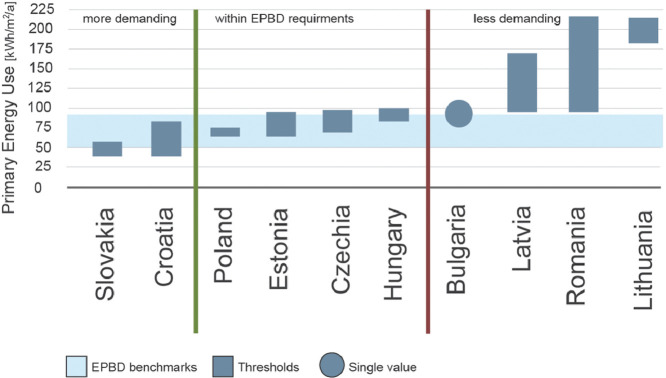

## Specifications Table

**Table:** 

Subject	Engineering
Specific subject area	Building energy performance.
Type of data	Excel sheets and pdf document representing Tables, images and data sheets.Table: Dataset01.xlsx
How the data were acquired	A literature review of restricted national publications and an extensive questionnaire with 14 national experts involved with the nZEB implementation plans and policies were conducted in 2021 and 2022.
Data format	Excel and PDF with data that was analyzed and compared.The data set comprises two types of data formats:RawAnalyzed
Description of data collection	Data of building performance requirements and market status were collected and compiled in tables. Data was collected from the ten countries and revised by 14 national experts. The dataset focused on 2015 to 2020 in Bulgaria, Croatia, Czech Republic, Estonia, Hungary, Latvia, Lithuania, Poland, Romania, and Slovakia.
Data source location	All sources all listed in the Excel and pdf files. Additionally, the data is externally deposited in a publicly available repository.
Data accessibility	Data are provided in supplementary materials directly with thisarticle. Additionally, the data is externally deposited in a publicly available repository.Repository Name: Harvard DataverseData Identification Number: 10.7910/DVN/ZYVNLQDirect URL to data: https://dataverse.harvard.edu/dataset.xhtml?persistentId=doi:10.7910/DVN/ZYVNLQS. Attia, ‘Data on residential nearly Zero Energy Buildings (nZEB) design in Eastern Europe’, *Harvard Dataverse*, vol. 1, 2022, doi:10.7910/DVN/ZYVNLQ. Accessible: https://dataverse.harvard.edu/dataset.xhtml?persistentId=doi:10.7910/DVN/ZYVNLQThe questionnaire can be found in the dataset above.
Related research article	[1] Attia, S., Kurnitski, L., Kosiński, P., Borodiņecs, A.,Belafi, Z.D., István, L., Krstić, H., Moldovan, M., Visa, I., Mihailov, N., Evstatiev, B., Banionis, K., Čekon, M., Vilčeková, S., Struhala, K., Brzoň, R., and Laurent, O. (2022) Overview and future challenges of nearly zero-energy building (nZEB) design in Eastern Europe, Energy and Buildings, 112,165,ISSN 0378–7788, https://doi.org/10.1016/j.enbuild.2022.112165.(https://www.sciencedirect.com/science/article/pii/S037877882200336X)

**Value of the Data**

• The data is valuable for the building energy efficiency research community, including building energy professionals, building energy systems inventors, and scientists who seek to build future scenarios for energy efficiency and carbon emissions. Scientists engaged in building science, architecture, mechanical engineering, and climate engineering are secondary research beneficiaries. More importantly, the data allows scientists to design energy policies that translate design data and code requirements for full electric, energy neutral, and carbon-neutral buildings.
• The data provide quantitative information on the design requirements of nearly Zero Energy Buildings (nZEBs) across Eastern Europe between 2015 and 2020.The provided data can be used to develop energy conservation measures and build energy models for energy loads in buildings and the future carbon emissions of residential buildings. Provided data account for European carbon neutrality targets.
• The data give insight into the implications of nZEB implementations on energy consumption, heating and cooling energy needs, thermal comfort, renewable production, and selected energy efficiency measures.

## 1 Data Description

This study collected data from fourteen stakeholders involved in the ten countries to develop nZEB performance requirements, classify them, and fuse them into shared data. The data is externally deposited in a publicly available repository [1]. Three files in the format of an Excel sheet and pdf document representing Tables, images and data sheets are available in the dataset.

### 1.1 Table: Dataset01.xlsx

Sheet 1: Legislation status in eastern Europe's member states in 2021.
Sheet 2: nZEB performance threshold in Eastern Europes’ Member States.
Sheet 3: Primary energy conversion factors in Eastern Europe's MS for the year 2020.
Sheet 4: Newly constructed households between 2015 and 2020 based on issued permits.
Sheet 5: Airtightness requirements for nZEB in the 10 EE countries.
Sheet 6: The energy modeling approach used for the nZEB calculation in EE countries.
Sheet 7: Mechanical ventilation requirements for nZEB in the ten countries.
Sheet 8: Share of fuels in the final energy consumption in the residential sector.
Sheet 9: Share of fuels in the final energy consumption in the residential sector for space heating, 2019 (%) Source: Eurostat.
Sheet 10: EPBD Cost optimality calculation approach and reference buildings.

**Fig. 1:**
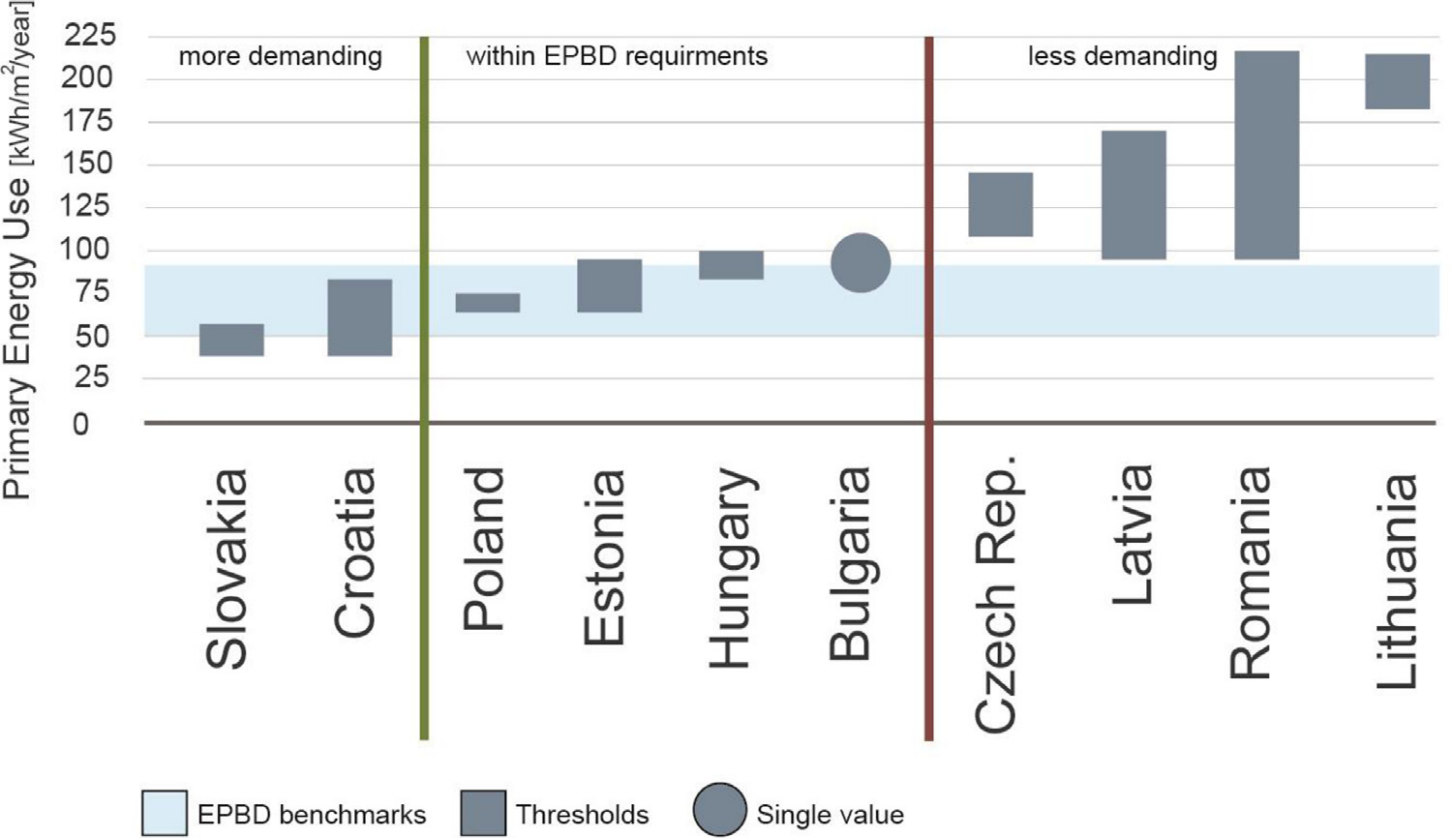
Comparing the nZEB requirements in single family residential buildings in the ten Eastern European countries.

An example of data visualization from the provided data files is shown in Fig. 1 and Table 1. Fig. 1 compares the primary energy use intensity requirements compared to the EPBD recommendations. Moreover, Table 1 provides the detailed performance requirements for nZEB single-family houses in the ten countries. The graph indicates the renewable energy shares requirements and the heating degree days and cooling degree-days in the major Eastern European cities.

**Table 1:** nZEB performance threshold in Eastern European countries, mainly for single-family households.

		Min. Energy Efficiency					2015–2020			
		Energy need for Cooling	Energy need for Heating	Primary Energy		Summer Climate	CDD	HDD		
Country	Climate Zone	kWh/m^2^/year	kWh/m^2^/year	kWh/m^2^/year	RES share	Cities	25 °C	20 °C	Latitude	Altitude
	1					Sofia	100	3476	42.65°N	550m
BULGARIA	2	None	None	95	No	Plovdiv	200	2823	42.13°N	164m
	3					Burgas	121	2646	42.50°N	30m
	1	None	None	50–80	No	Zagreb	400	2892	48.51°N	122m

CROATIA	2					Split	788	1749	43.3°N	12m
	1	reference building	estimation 110–130		Prague	115	3439	50.5°N	235m	

CZECH R.*	2			No	Brno	135	3198	49.11°N	237m	
	3				C. Budejovice	51	3658	48.58°N	381m	

ESTONIA	1	None	None	100–145	No	Tallinn	2	5274	59.4°N	33m

HUNGARY	1	None	None	85–100	No	Budapest	497	3070	47.49°N	98m
	2					Pécs	501	2969	46.07°N	153m

LATVIA	1	None	40–60	95–170	No	Riga	22	3846	56.94°N	2m

LITHUANIA⁎⁎	1		35–75	180–220	>50%	Vilnius	31	4852	54.6°N	156m
	1					Warsaw	58.7	2972	52.2°N	99m
	2					Olsztyn	18.1	3322	53.8°N	121m

POLAND	3	None	None	65–75	No	Wroclaw	53.9	2847	51.1°N	131m
	4					Gdansk	12.9	3234	54.3° N	8m
	5					Suwalki	13.0	2484	54.1°N	170m

ROMANIA	1	70	20	93–217	>30%	Bucharest	343	2075	44.4°N	77m
	2					Cluj-Napoca	16	2976	46.7°N	380m
	3					Brasov	1	3112	45.6°N	610m

SLOVAKIA	1	None	None	32–54	No	Bratislava	184	2964	48.8°N	134m
	2					Prešov	184	3574	49.0°N	250m

*Note:* The baselines applied for the HDD, and CDD calculation is 20 and 25 °C data from 2015 to 2020 was used. The hourly approach shows a significant CDD change regarding day/night variation compared to the HDD.

⁎ **CZECH REPUBLIC**: There are no particular values or thresholds according to ordinance no. 264/2020 Coll. A comparison with reference building is used. Energy need for heating: ≤ 1.0 * E_R_  (E_R_ = reference building) and – Primary Energy: ≤ 1.6 * E_R_ (E_R_ = reference building).

⁎⁎ **LITHUANIA:** Energy need for heating: 100 m^2^ – 75 kWh/m^2^, 200 m2 – 57 kWh/m^2^, 2000 m^2^ – 35 kWh/m^2^ – Primary Energy: 100 m^2^ – 220 kWh/m^2^/year, 200 m^2^ – 190 kWh/m^2^/year, 2000 m^2^ – 180 kWh/m^2^/year.

### 1.2 Report: Dataset03.pdf

The report files comprises the above mentioned tables and figures in the Excel Sheet (Table: Dataset01.xlsx). The report describes each table and figure in detail.

### 1.3 Questionnaire: Questionnaire.pdf

The file lists the questionnaire used during the data collection stage of the experts.

## 2 Experimental Design, Materials and Methods

The European Union is looking to neutralize the carbon emissions of the building and construction sector by 2050. The Fit to 55 legislation package seeks to achieve carbon-neutral buildings by 2030 across Europe [2]. The Energy Performance of Building Directive is the instrument that is used by the European Commission to translate the carbon neutrality targets into technical plans and requirements for the newly built and existing buildings [3]. In this context, nearly and net-zero energy buildings, which use ultra-low energy annually and compensate for the energy use through onsite or nearby renewable energy [3], ensure reaching this goal [4]. Nearly zero-energy buildings (nZEBs) play a role and are considered the first milestone towards net-Zero Energy Buildings (nZEB) and nearly zero carbon buildings (nZCB) [5]. Since 2018, many advances have been reported in Western Europe [6] and Southern Europe [7]. However, very little information is available regarding the market uptake and legislation progress of nZEB in Eastern Europe. Eastern Europe is a vital continent with steadily growing economies that must be properly studied concerning the built and renovated building stock [8]. The geopolitical situation and the history of Eastern Europe go beyond decarbonization of heating energy use. It is subject to building construction quality, indoor environmental quality, renewable energy, and standards. Although Eastern European countries report the progress of nZEB concept adoption, there is a need for updated and reliable data that characterizes the situation and implementation of nZEB requirements in new building standards and practice across Eastern Europe [9]. The state of nZEB plans implementation (e.g. requirements of heating and cooling energy loads thresholds and the associated greenhouse gas emissions limits and the mandatory requirements for renewable energy production shares) is not fully known.

This study collected data from sixty reviewed documents and fourteen stakeholders involved in the ten countries to develop nZEB performance requirements, classify them, and fuse them into shared data. The data identify the performance requirements and construction assurance requirements through qualitative and quantitative variables. The methodology is based on an iterative approach of data collection and verification, where literature reviews and experts questionnaires are used to compare the reported facts.

Firstly, the methodology reviewed data available on the different European platforms such as the European Commission (EC) Eurostat, European Construction Sector Observatory (ECSO), Building Performance Institute Europe (BPIE), ODYSSEE database on energy efficiency indicators and energy consumption by end-use and their underlying drivers in buildings. And national energy agencies in the ten investigated countries were reviewed. Double checks and reviews took place to make sure the data was valid. Then, the data was sorted and grouped in tables and visualized in figures to cross-comparison between the countries.

The second step of the methodology is an experts-based questionnaire – a long questionnaire with seven main questions and twenty-three sub-questions allowed to collect specific information on nZEB requirements. The collected information includes climate characterization, minimum energy efficiency, heating/cooling energy needs, thermal comfort limits, renewable energy needs, and construction quality and inspection measures. The survey questions are provided in the data files included the compiled experts' answers. Among the collected data, the following questions provide an overview of the provided information:

• nZEB definitions and carbon emissions requirements;
• minimum energy efficiency threshold;
• envelope performance requirements;
• heating-cooling balance and systems efficiencies;
• mechanical ventilation systems requirements and specifications;
• thermal comfort models, standards, and thresholds;
• overheating calculation approach;
• simulation approach;
• energy performance certificate requirements;
• renewables energy systems and production share onsite or nearby;
• construction quality and performance inspection measures.

The shared data include both the questions' raw answers and refined and processed data in the form of comparative Tables and Figures.

*Recruitment process***:** Building energy efficiency experts were identified based on a literature review screening. Most of the selected experts represented building physics engineers, mechanical engineers, and architects involved in the national standards development. All interviewed experts had experiences ranging from 6 to 15 years and had worked in national EPBD development. The recruitment continued until we reached saturation and heterogeneity.

The continuation of the interviewee recruitments was based on data saturation and interviewee homogeneity. The recruitment processes started in 2020 and ended in summer 2021when insights and answers started to replicate and overlap. Data saturation was used as a factor to judge the repetition of answers by the expert that indicated similar relevant insights.

*Validation***:** Qualitative research is primarily subjective as it seeks to understand human perceptions and judgments. However, qualitative research remains the most powerful exploratory scientific method that provides valuable insights and interpretations when bias is avoided. Therefore, validating the questionnaires' is essential to provide reliable and consistent results. The data went through several validation rounds. The study validation emphasized credibility and strengthened the accuracy of the collected data. The following measures were applied to collect and interpret valid information.

First, member checking was used to explore the credibility of the results. The experts had to revise and approve their questionnaire answers after transcription. Every questionnaire was returned to the participants to check for accuracy and supported with references.

Second, peer examination of the input data was achieved by involving a second expert for the same country. We recruited secondary experts from other national institutions or universities. This provided a second opinion on the coding process to ensure higher credibility for our research outcomes.

Thirdly, the prolonged engagement of spending more than 2years (2020–2022) in the field helped us learn or understand the legislative and technical setting of nZEB. Many fact checks and cross-referencing based on the literature review took place with the help European Commission (EC), Eurostat, European Construction Sector Observatory (ECSO), Building Performance Institute Europe (BPIE), ODYSSEE database on energy efficiency indicators and energy consumption by end-use and their underlying drivers in buildings to validate the data.

## Supplementary Material

The data is externally deposited in a publicly available repository [1].

## Ethics Statements

The above works do not contain information from human subjects, animal experiments or data collected from social media platforms.

## CRediT authorship contribution statement

**Shady Attia:** Conceptualization, Methodology, Investigation, Data curation, Formal analysis, Validation, Supervision, Visualization, Writing – original draft, Writing – review & editing.

## Declaration of Competing Interest

The authors declare that they have no known competing financial interests or personal relationships that could have appeared to influence the work reported in this paper.

## Data Availability

Data on residential nearly Zero Energy Buildings (nZEB) design in Eastern Europe (Original data) (Dataverse).
